# AtCRK5 Protein Kinase Exhibits a Regulatory Role in Hypocotyl Hook Development during Skotomorphogenesis

**DOI:** 10.3390/ijms20143432

**Published:** 2019-07-12

**Authors:** Abu Imran Baba, Norbert Andrási, Ildikó Valkai, Teréz Gorcsa, Lilla Koczka, Zsuzsanna Darula, Katalin F. Medzihradszky, László Szabados, Attila Fehér, Gábor Rigó, Ágnes Cséplő

**Affiliations:** 1 Institute of Plant Biology, Biological Research Centre, Hungarian Academy of Sciences, 6726 Szeged, Hungary; 2 Doctoral School in Biology, Faculty of Science and Informatics, University of Szeged, 6720 Szeged, Hungary; 3 Agricultural Biotechnology Institute, Szent-Györgyi Albert u. 4, H-2100 Gödöllő, Hungary; 4 Developmental and Cell Biology of Plants, CEITEC Masaryk University, 62500 Brno, Czech Republic; 5 Department of Plant Biology, University of Szeged, 52. Közép fasor, H-6726 Szeged, Hungary

**Keywords:** Ca^2+^/calmodulin-dependent kinase-related kinases (CRKs), polar auxin transport (PAT) proteins, auxin gradient, ethylene, GA_3_, skotomorphogenesis, *Arabidopsis thaliana*

## Abstract

Seedling establishment following germination requires the fine tuning of plant hormone levels including that of auxin. Directional movement of auxin has a central role in the associated processes, among others, in hypocotyl hook development. Regulated auxin transport is ensured by several transporters (PINs, AUX1, ABCB) and their tight cooperation. Here we describe the regulatory role of the *Arabidopsis thaliana* CRK5 protein kinase during hypocotyl hook formation/opening influencing auxin transport and the auxin-ethylene-GA hormonal crosstalk. It was found that the At*crk5-1* mutant exhibits an impaired hypocotyl hook establishment phenotype resulting only in limited bending in the dark. The At*crk5-1* mutant proved to be deficient in the maintenance of local auxin accumulation at the concave side of the hypocotyl hook as demonstrated by decreased fluorescence of the auxin sensor DR5::GFP. Abundance of the polar auxin transport (PAT) proteins PIN3, PIN7, and AUX1 were also decreased in the At*crk5-1* hypocotyl hook. The AtCRK5 protein kinase was reported to regulate PIN2 protein activity by phosphorylation during the root gravitropic response. Here it is shown that AtCRK5 can also phosphorylate in vitro the hydrophilic loops of PIN3. We propose that AtCRK5 may regulate hypocotyl hook formation in *Arabidopsis thaliana* through the phosphorylation of polar auxin transport (PAT) proteins, the fine tuning of auxin transport, and consequently the coordination of auxin-ethylene-GA levels.

## 1. Introduction

Seedlings of dicotyledonous plants develop an apical hook in the dark following germination in order to avoid the mechanical damage of the apical meristem when emerging from under the soil [1,2,3,4]. Apical hook formation is generated by differential cell elongation at two sides of the hypocotyl apex [3,5,6,7]. The apical hook development consists of three successive phases, the formation, maintenance and opening phases. The typical kinetics of these apical hook formation phases has been determined in Arabidopsis as 0–54 h, 54–90 h and 90–120 h after germination, respectively [5,6,7]. The formation of the apical hook is basically regulated by the phytohormone auxin [2,3,4,8,9,10,11,12,13] which gradually accumulates at the concave side of the hook during hook development and inhibits cell elongation [5,6]. The auxin concentration remains constant upon the maintenance phase resulting in closed hook [5,6]. When this asymmetrical auxin gradient gradually disappears, then the hook enters into the opening phase which is naturally triggered by light [10,11].

In Arabidopsis, auxin is transported by the PIN-FORMED (PIN) efflux transporters, the AUX1/ LIKE-AUX1 (AUX/LAX) auxin influx protein family and the ABCB transporter superfamily members (PGP proteins) [14,15,16,17,18,19]. The directional movement of auxin is ensured by the polar subcellular localization of these transporters [20]. Amongst the eight Arabidopsis PIN proteins, PIN3, PIN4 and PIN7 are expressed during hypocotyl hook formation [5,6]. The asymmetric accumulation of auxin is regulated by two hormonal pathways influencing also each other, the gibberellin-dependent DELLA-PIF (PHYTOCHROME INTERACTING FACTORs) and the ethylene-controlled EIN3/EIL1 (ETHYLENE-INSENSITIVE 3/EIN3 like 1)–HLS1 (HOOKLESS 1) pathways [11]. The complex interplay amongst the elements of this hormonal network is fundamental to control the establishment of the normal hypocotyl hook during skotomorphogenesis [10,11,21].

Elevated ethylene content or ethylene mutants with upregulated signaling (*eto1* or *ctr1*) result in exaggerated apical hook of etiolated Arabidopsis seedlings [22,23]. In agreement, the ethylene insensitive mutants *etr1* and *etr2* are hookless [8,22]. Ethylene is able to regulate auxin function in many ways in order to promote hook development [10,11]. Ethylene promotes the expression of the TRYPTOPHAN AMINOTRANSFERASE2 (*TAR2*) gene resulting in increased auxin level in the apical hook [5]. Ethylene promotes the expression of HOOKLESS1 (*HLS1*) encoding a putative N-acetyltransferase [24] inhibiting the expression of AUXIN RESPONSE FACTOR2 (ARF2), a repressor of auxin signaling [9,11]. Ethylene modulates the turnover of AUX1 in the inner side of the hook [5] and also affects the expression of several *PIN* genes and the localization of PIN3 [6]. Ultimately, ethylene positively influences auxin responses in the hypocotyl hook.

Gibberellins have also prominent role in hypocotyl hook development by fine tuning the regulation of auxin-ethylene levels [10,11]. DELLA proteins are negative regulators in the GA signaling pathway in Arabidopsis [10,25,26] and quintuple mutant seedlings knocked out for five DELLA protein genes exhibit an exaggerated hook, whereas seedlings with overexpressed DELLA proteins enter immediately into the opening phase of hook development [21,26]. GAs are required for the expression of PIN3 and PIN7 auxin efflux carriers which are downstream of GA since the *pin3 pin7* mutant does not show exaggerated curvature following exogenous GA application [7]. The molecular mechanism by which GAs control the expression of the above auxin transporters is not known. Furthermore, GAs regulate the expression of the AGC-type kinase WAG2 controlling the localization of PINs likely via their phosphorylation [27]. WAG2 is preferentially expressed at the inner side of the hook contributing to asymmetric auxin action and thus preventing hook opening. It is also known that auxin has a promoting effect on the expression of gibberellin biosynthesis and transport genes resulting in a positive feedback loop during hook development [28,29,30,31]. Altogether, GAs and ethylene cooperatively prevent apical hook opening acting on auxin synthesis, transport [7].

The Calcium-Dependent Protein Kinases (CDPKs) are among the main regulators in Ca^2+^ signaling [32,33,34,35,36]. The Ca*^2+/^*Calmodulin-Dependent Protein Kinase-Related Kinases (CRKs) are Ser/Thr protein kinases, which have diverse regulatory functions in plant growth and development, abiotic and biotic stress responses and in phytohormone regulation [37,38]. A unique feature of the CDPKs/CRKs superfamily is their N-terminal myristoylation site, suggesting that these proteins are localized at the plasma membrane or other endomembranes of other organelles like endoplasmic reticulum, tonoplast, mitochondria, chloroplasts, oil bodies, peroxisomes and Golgi network [39,40,41]. Functionality of most plant CDPKs are well characterized, however, only a few CRKs have been characterized by their biological activities in angiosperms [37,38,42]. The Arabidopsis CRK subfamily consists of eight members [32,33,34,35,36]. Interestingly, some of the AtCRKs, in addition to their Ser/Thr phosphorylation capability, were claimed to have Tyr kinase activity as well [43]. The AtCRK5 protein kinase was previously characterized in our laboratory. Intracellular localization pattern of AtCRK5-GFP fusion protein in roots displayed distribution of this protein at the plasma membrane [42]. This localization pattern is indicative for that proteins which are involved in nutrient uptake, as it was observed e.g., for boron transporters in Arabidopsis [44]. The confirmation of regulatory role of AtCRK5 protein kinase in microelement uptake as well as in water transport regulation requires further studies. The first biological function discovered for AtCRK5 was that this protein kinase had a direct role in the regulation of root gravitropic response [42]. Impaired gravitropic response was a consequence of altered auxin distribution in the At*crk5-1* mutant root tips as compared to the wild type. Immunolocalization pattern of the auxin efflux protein PIN2—which is a key member of basipetal auxin transport in Arabidopsis roots—exhibited a considerable alteration in the At*crk5-1* mutant in comparison to the wild type [37,42]. The AtCRK5 protein kinase phosphorylates the PIN2 auxin efflux protein in vitro [42]. Phosphorylation of PIN2 in At*crk5-1* mutant roots is impaired, which hinders and ultimately delays the establishment of the asymmetrical auxin gradient basically required for normal root bending upon gravistimulation [42]. After highlighting the regulatory role of AtCRK5 in root gravitropism, we also observed that germinating in the dark, the At*crk5-1* mutant seedlings had a decreased apical hook angle through all hook developmental phases in comparison to the apical hooks of the wild type. Similar impaired apical hook development phenotype was described for the At*wag2* mutant [27]. However, how the auxin maxima at the hypocotyl hook are influenced by coordinated actions of the various auxin transporters and other regulatory factors (e.g., protein kinases) is still poorly understood. Based on the above observations, the potential regulatory role of the AtCRK5 kinase in the formation and maintenance of the differential auxin gradient in the apical hook of the hypocotyl had been investigated during skotomorphogenesis.

Here we further demonstrate the importance of the AtCRK5 protein kinase in Arabidopsis growth and development. It seems that this protein kinase - additionally to its role in regulating root gravitropic response – also participates in the regulation of hypocotyl hook development during skotomorphogenesis. As a potential mechanism, the AtCRK5-mediated phosphorylation of the PIN3 auxin efflux transporter influencing auxin accumulation and the effect of limited auxin accumulation on ethylene and/or GAs action is discussed.

## 2. Results

### 2.1. Hypocotyl Hook Bending Angle Differences between Col-0 and Atcrk5-1

**Figure 1:**
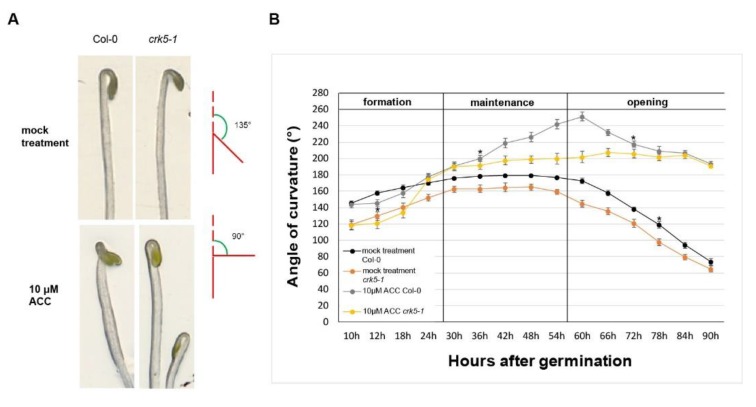
Hypocotyl hook phenotypes and kinetics of apical hook development of wild type (Col-0) and mutant (At*crk5-1*) seedlings during dark germination. (**A**) Hypocotyl hook phenotypes of 3-days-old dark-grown seedlings of the wild type (Col-0) and mutant (At*crk5-1*) in the absence and presence of 10 μM ACC. The wild-type seedlings had 180° hypocotyl hook bending, while the mutant seedlings displayed less hook bending capacity under normal conditions at 3 days after germination (during the hook maintenance phase). 10 μM ACC increased the bending of wild-type hooks and restored that of the At*crk5-1* mutant to app. 180°. The angle of hook curvature was determined by measuring the outer angle between the main axis of hypocotyl and curvature of cotyledons (see illustration). (**B**) Kinetics of apical hook development in Col-0 and At*crk5-1* dark-grown seedlings in the absence and presence of ACC. Averages with standard errors are shown (*n* = 35). Differences between the wild type and the mutant were statistically significant between the two indicated (*) time points (Student’s *t*-test: *P* < 0.01). Col-0 = At wild type Columbia-0 seedlings, At*crk5-1* = mutant seedlings. Two independent experiments were carried out with the same results.

Our initial observation was that 3-days-old At*crk5-1* seedlings had an altered phenotype as compared to the wild type ones (Col-0): the At*crk5-1* mutant exhibited a decreased capacity in the closure of the apical hook during skotomorphogenesis. Germinating the wild type seeds in the dark, normal hypocotyl hook formation was obtained with an angle of approximately 180° at 3 days, but the At*crk5-1* mutant seedling hook angles were smaller (145°–160°) under the same conditions (Figure 1A). Considering the role of ethylene in the inhibition of hook opening, we checked the influence of the ethylene precursor 1-aminocyclopropane-1-carboxylic acid (ACC) [45] upon hypocotyl development of the mutant in the dark. Following 10 μM ACC treatments, the wild type Col-0 seedlings showed the typical exaggerated hook phenotype (225°–240°), while the At*crk5-1* seedlings exhibited apical hook angles of 180°–200° (Figure 1B).

Since during seedling development in dark the ethylene sensitivity is restricted to the 2-3th days after germination [5,8,10] we compared the kinetics of the apical hook development in the *Arabidopsis thaliana* wild type Col-0 and At*crk5-1* mutant seedlings under normal (without ACC) and ethylene precursor ACC-treated conditions (Figure 1B).

**Figure 2:**
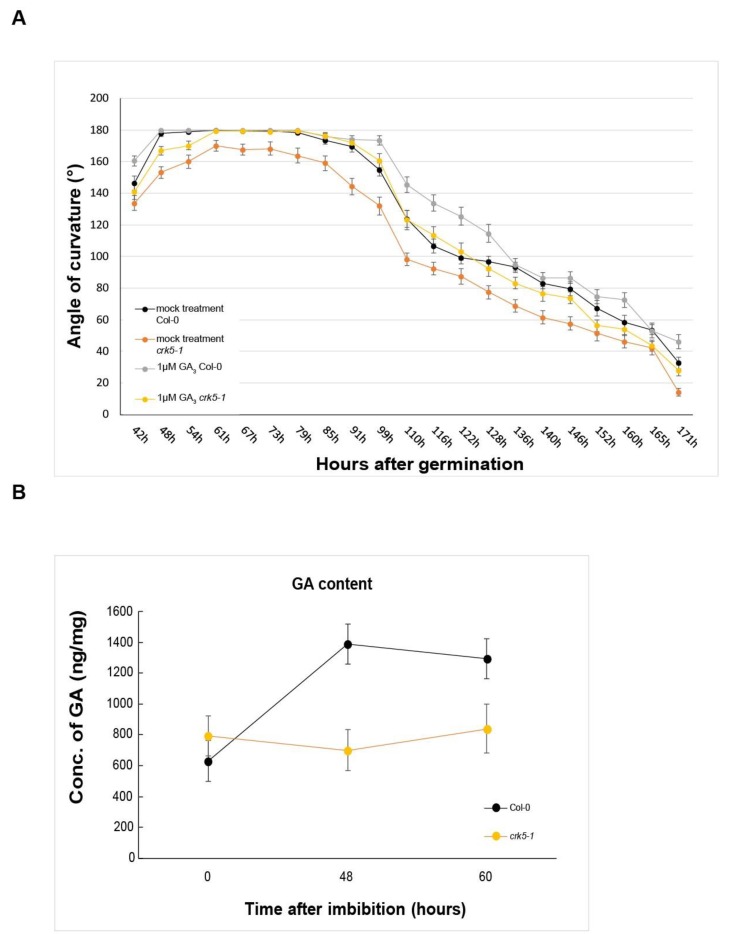
Kinetics of exogenous GA_3_-regulated apical hook development and measurement of the total GA content. (**A**) Kinetics of hypocotyl hook development in wild type Arabidopsis (Col-0) and in mutant At*crk5-1* seedlings germinated in dark in the absence and presence of 1 μM GA_3._ The experiments were carried out twice with the same results. The averages and standard error (SE) are shown of two biological replicates using minimum 35 seedlings per genotype per experiment. The mutant values are significantly different at all the time period tested in comparison with the wild type (Student’s *t*-test: *P* < 0.01). (**B**) Determination of total gibberellic acid concentrations in seeds and dark-grown seedlings of the wild type (Col-0) and the mutant (At*crk5-1*). Total GA content was measured in dried seeds (0 h), and in seedlings at hook formation (48 h) and at the beginning of the hook maintenance phase (60 h). Two biological repeats were evaluated.

Kinetics of the apical hook development was followed for 90 h in the dark. The three developmental (formation, maintenance and opening) stages of hook development were confirmed [8]. For the wild type Arabidopsis Col-0, similar hook kinetic results were obtained under normal condition and ACC treatment, respectively, as it was previously described [5,6,7]. In comparison to the wild type, the At*crk5-1* mutant showed decreased apical hook angle under normal conditions through the investigated period of hook development (see also Figure 2A). However, the kinetics of hook development (timing of the phases) was not affected by the mutation. The ethylene precursor ACC treatment delayed the transition between the formation and maintenance phases in the wild type as well as the At*crk5-1* seedlings leading to hooks with exaggerated curvatures; up to 240° in the wild type and 200° in the mutant background. Interestingly, opening of the hook was considerably delayed in the mutant in comparison of the control in the presence of ACC but not under normal conditions (Figure 1B).

From these data, one can conclude that the At*crk5-1* mutant has a very early apical hook formation phenotype indicating that AtCRK5 can contribute in some ways to the establishment of the required auxin gradient in Arabidopsis. Later phases seem to be unaffected by the mutation under normal conditions as indicated by the parallel kinetics of hook development in wild-type and mutant backgrounds. The kinase mutant responded to exogenous ACC by hypocotyl bending, while in the absence of the kinase, ACC application not only delayed but prevented the transition from the maintenance to the opening phase (Figure 1B). These observations indicate that the kinase is not required for the curvature-inducing but prevents the opening-inhibitory effect of ethylene during hook development.

### 2.2. Kinetics of GA_3_-Regulated Apical Hook Development

During the formation and maintenance phases, another hormone, gibberellin (GA_3_) also performs a major role in hypocotyl hook development of dicots (7, 10). According to [7], the GA pathway is required to reach the threshold level of ethylene that is capable to postpone hook opening in wild type seedlings. Therefore, we performed time lapse experiments to investigate the effect of GA_3_ on hypocotyl hook formation in the dark. Figure 2A shows the kinetics of hypocotyl hook development in dark-germinated wild-type Col-0 and mutant At*crk5-1* seedlings in the absence and presence of 1 μM GA_3_, respectively.

Exogenous GA_3_ did not affect the angle of the hooks of wild-type seedlings in the maintenance phase. The At*crk5-1* mutant, however, responded to exogenous GA_3_ with increased hypocotyl bending during the maintenance phase followed by slightly faster opening than in the wild type (Figure 2A).

Measurement of the total GA content of the seedlings at the hook formation and maintenance phases displayed considerable differences between wild type Col-0 and At*crk5-1* genotypes. While in dried seeds the total GA concentration was nearly similar in both cases, at the beginning of germination (48h and 60h after germination of seeds) we found two-fold less total GA concentrations in the At*crk5-1* mutant than in the wild type seedlings (Figure 2B).

It is known that there is a positive feedback loop between auxin and gibberellin because auxin has a promoting effect on expression of gibberellin biosynthesis genes [28,29,30,31]. Therefore decreased auxin level may negatively influence the GA biosynthesis resulting in lower GA content as it was found in the case of the dark germinating At*crk5-1* seedlings. However, exogenous GA_3_ could restore the At*crk5-1* mutant hypocotyl hook bending phenotype to the wild type level at the maintenance phase of germination indicating a cooperative action of these two hormones on hypocotyl hook development.

### 2.3. AtCRK5 is a Regulator of the Auxin Maxima in the Apical Hook

Previous mutant studies of the auxin efflux facilitators PIN1, PIN3, PIN4 and PIN7 indicated that these proteins are the main players during the formation of the apical hook [6,19,27]. Amongst these, the At*pin3* mutant has the most prominent apical hook phenotype indicating that PIN3 is the most important auxin carrier in this process. PIN3 acting mainly at the outer side of the hook, distributes auxin from the vascular tissue into the cortex and epidermis as well as through these tissues down the hypocotyl [6]. However, other auxin influx carriers like AUX1/LAX3 are also necessary to direct the auxin flow during hypocotyl hook development [5,10]. The AtCRK5 protein kinase was previously described as a regulator of PIN2 function during root gravitropic responses [37,42]. In order to test that there is a link between AtCRK5 function and auxin transport regulation also during the formation of hypocotyl hook, we studied the distribution patterns of the auxin response marker DR5::GFP and the GFP tagged auxin transporters PIN3, PIN7 and AUX1-YFP proteins in 3-days-old dark-grown wild type and mutant seedlings without and with ACC/GA_3_ treatments.

#### 2.3.1. Distribution of the Auxin in Hypocotyl Hooks monitored by DR5::GFP Fluorescence

**Figure 3:**
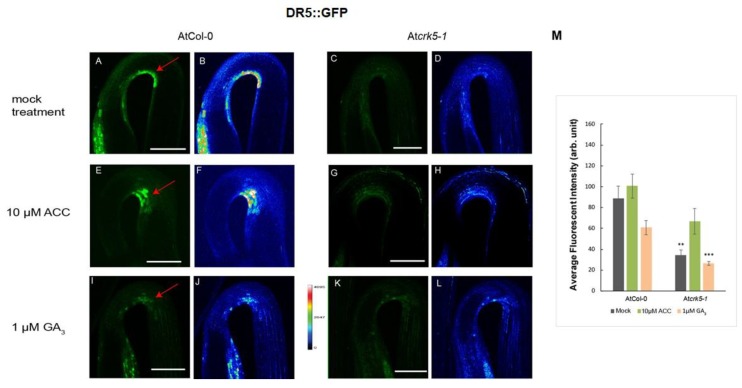
AtCRK5 regulates the hypocotyl hook auxin maximum. Distribution of the DR5::GFP signal in 3- days-old dark-grown Col-0 wild-type and mutant At*crk5-1* seedlings treated with 10 μM ACC or 1 μM GA_3_. DR5::GFP signal is located at the concave side of the hypocotyl hook in the wild type (**A**,**B**) and it is much stronger than in the At*crk5-1* seedling (**C**,**D**). Exogenous ACC enhances the DR5::GFP signal in the apical hook in the wild type (**E**,**F**) as well as in the mutant hooks (**G**,**H**). Red arrows point the GFP signals in the hook region. 1μM GA_3_ treatment broadened the DR5::GFP signal at the apical hook of the wild type (**I**,**J**) and mutant (**K**,**L**). Figure 3B–D, F–H and J–L indicate the corresponding GFP signal intensity heat maps for the wild type and mutant, respectively. (**M**) Relative intensity of the DR5::GFP signals at the concave side of the hypocotyl hooks. 10 seedlings from wild type and mutant categories were investigated in each version. Asterisks indicate significant differences, compared with the corresponding Col-0 mock control and treatments (Student’s *t-*test, ** *P* < 0.001, *** *P* < 0.0001). All experiments were repeated for two times. Scale bars = 200 μm.

First, we examined the auxin distribution in the apical hook region of the wild type Col-0 and mutant At*crk5-1* seedlings using the auxin response reporter construct DR5::GFP (green fluorescence protein) [46]. Expression studies of the DR5::GFP revealed that the dark-grown wild-type Arabidopsis seedlings have strong DR5::GFP signal in the concave part of the hypocotyl hook (Figure 3A) contrary to the At*crk5-1* seedlings which have very faint DR5::GFP signal in that region (Figure 3C) indicating inadequate establishment of the auxin gradient. GFP signal intensities are represented via heat maps for the wild type (Figure 3B) and for the mutant (Figure 3D). Quantification of the fluorescence signals is in Figure 3M. Addition of 10 μM ACC elevated and expanded the GFP signal intensity in both cases (Figure 3E for wild type and Figure 3G for mutant seedlings), especially in the At*crk5-1* hook which is well supported by heat map images (Figure 3F for the wild type and Figure 3H for the mutant) and the quantitative data (Figure 3M). This suggests that exogenous ethylene reinforced the asymmetrical auxin gradient as described in [47]. Treatment with 1μM GA_3_ rather broadened the area than enhanced the intensity of the GFP signal both in the wild type (Figure 3I) and the mutant At*crk5-1* (Figure 3K) which is also well demonstrated by heat maps (Figure 3J for the wild type and Figure 3L for the At*crk5-1* mutant) and quantification of the fluorescence intensities (Figure 3M).

#### 2.3.2. Distribution of PIN3-GFP, PIN7-GFP and AUX1-YFP in Hypocotyl Hooks

**Figure 4:**
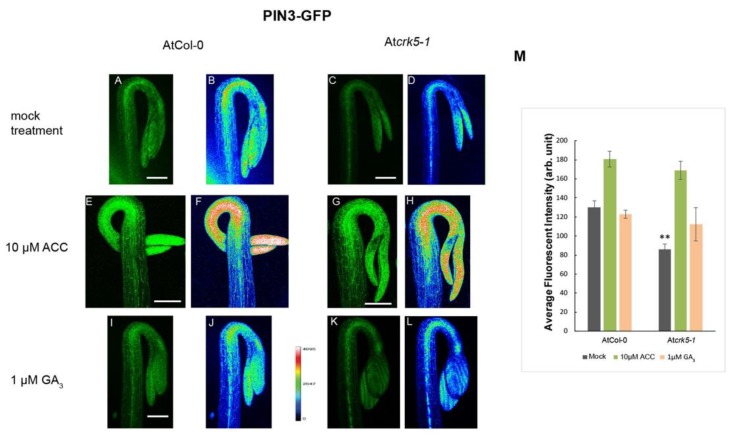
Distribution of the PIN3-GFP signals in hypocotyl hooks. 3- days-old dark-grown wild type and mutant seedlings were treated with 10 μM ACC and 1 μM GA_3_, respectively. PIN3-GFP signal is more intense in the wild type stele (**A**,**B**) than in that of the At*crk5-1* mutant (**C**,**D**). Exogenous ACC strongly enhanced and broadened the PIN3-GFP signal in the apical hook both in the wild type (**E**,**F**) and the mutant (**G**,**H**). GA_3_ treatment extended the PIN3-GFP signals in the wild type (**I**) and mutant (**K**) hooks. Heat map images (**B**-**F**-**J** for wild type and D-H-L for mutant) are also shown. (**M**) The relative intensities of the PIN3-GFP signals measured between the convex and concave sides of the wild type and mutant hooks. 10 optically sliced seedlings from wild type and mutant categories were used for quantification in each treatment. Double asterisks indicate *P* < 0.001. (Student’s *t-*test, compared with the corresponding mock control). All experiments were repeated two times. Scale bars = 200 μm.

**Figure 5:**
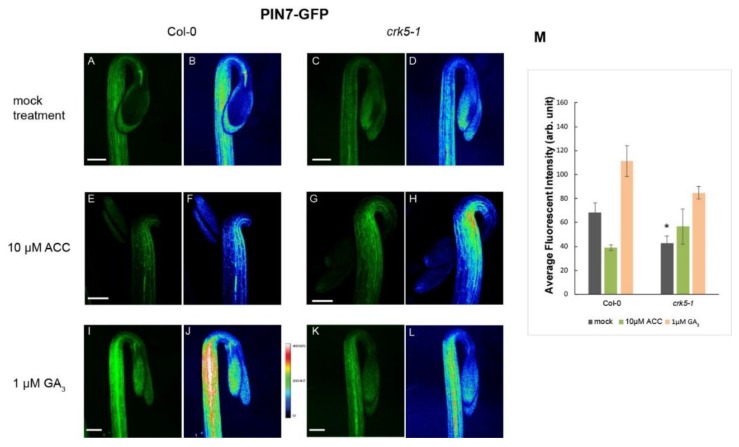
Abundance of the PIN7-GFP signal in hypocotyl hooks. PIN7-GFP signal is much stronger in the wild type (**A**,**B**) than in 3 days old dark grown At*crk5-1* mutant background (**C**,**D**). Upon ACC treatment, the PIN7-GFP signal is dispersed in wild type (**E**,**F**) and mutant (**G**,**H**) apical hooks. Exogenous GA_3_ strongly enhances the PIN7-GFP signal in the Col-0 wild type (**I**,**J**) and even more in the mutant (**K**,**L**) too. (**M**) The relative intensity of the PIN7-GFP signals measured in the hypocotyls of the wild type and mutant. Single asterisk indicates *P* < 0.05 (Student’s *t-*test, compared with the corresponding mock control; *n* = 10 seedlings per treatments). All experiments were repeated two times. Scale bars = 200 μm.

**Figure 6:**
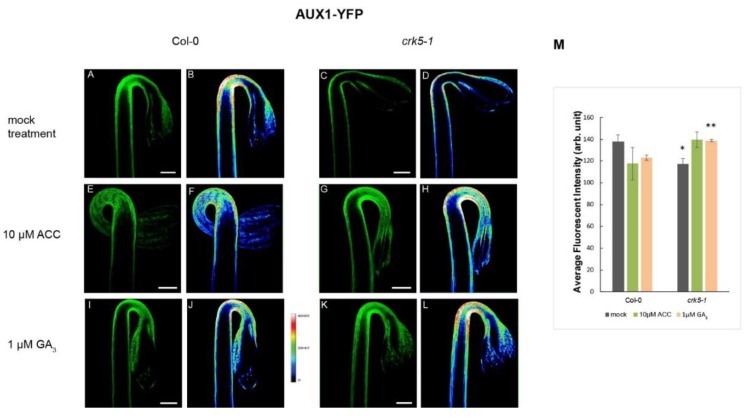
Distribution of the AUX1-YFP signals in 3-days-old dark-grown Col-0 wild-type and At*crk5-1* mutant seedlings. Abundance of the AUX1-YFP signal in the hypocotyl hook of Col-0 wild type (**A**,**B**) and the mutant At*crk5-1* (**C**,**D**). There is significantly lower YFP signal in the mutant hook (**C**,**D**) than in that of the wild type (**A**,**B**). Upon ACC treatment, the YFP signal became extended in both categories (**E**,**F** and **G**,**H**) and intensified in the mutant hook (**G**,**H**). GA_3_ treatment broadened the AUX1-YFP signal area at the apical hook of the wild type (**I**,**J**) and mutant (**K**,**L**) and elevated the mutant YFP signal intensity to the wild type level (**K**,**L**). (**M**) Relative intensity of the AUX1::YFP signals in hypocotyl hooks. Asterisks indicate significant differences, comparing the mutant to the corresponding Col-0 control (Student’s *t-*test, * *P* < 0.05 and ** *P* < 0.001, *n* = 10 seedlings per experiment). All experiments were repeated two times. Scale bars = 200 μm.

It has been described that ethylene is able to modulate the auxin transport in the hypocotyl hook enforcing the preferential localization of PAT proteins, especially the PIN3 location to the lateral side of cortex cells mainly at the outer side of the hook [6,13]. We tested the requirement of AtCRK5 for proper PAT proteins PIN3, PIN7 and AUX1 localization during hypocotyl hook formation in the dark. Distribution of the PIN3-GFP (PIN3:PIN3-GFP) [6] revealed that PIN3 proteins are located in the central cylinder of the hypocotyl hook of the wild type seedlings (Figure 4A,B) and it is less intense in the At*crk5-1* mutant (Figure 4C,D) seedlings, and the GFP signal was less intense in the mutant compared to Col-0 (Figure 4M). PIN7-GFP (PIN7:PIN7-GFP) [48] signal is located at the hypocotyl in the wild type Arabidopsis [15,17]. We found that the PIN7-GFP signal is less intense in the At*crk5-1* mutant (Figure 5C,D) as compared to that in the wild type Col-0 (Figure 5A,B). We also examined the auxin influx AUX1-YFP (AUX1::YFP) [49] distribution pattern in the apical hook of the At*crk5-1* mutant during dark germination. AUX1-YFP is localized mainly in epidermal cells of the hook at both sides and participates in the auxin flow from cotyledons towards the lower part of hypocotyl [5,10,12]. We observed that there is a significant YFP signal intensity decrease in the mutant apical hook (Figure 6C,D) when compared to those of the wild type (Figure 6A,B). Alterations in signal intensity are also shown by heat maps.

Exogenous ACC enhanced and broadened the PIN3-GFP signal in the wild type (Figure 4E,F) and in the mutant (Figure 4G,H) hooks. ACC-induced broadening of the PIN7-GFP signal was also observed in the apical hook of both genotypes (Figure 5E,F and Figure 5G,H). External ACC again rather extended the area then intensified the AUX1-YFP signal in wild type (Figure 6E,F) and in the mutant (Figure 6G,H) apical hooks. Signal intensity changes are represented by heat maps.

Upon 1 μM GA_3_ treatment, the PIN3-GFP signal became extended both in the wild type (Figure 4I,J) and the mutant (Figure 4K,L) hypocotyl hooks. External GA_3_ strongly elevated the PIN7-GFP signal intensity which became more dispersed in both phenotypes (Figure 5I,J for wild type and Figure 5K,L for the mutant). In case of AUX1-YFP, the GA_3_ treatment seemed to have a stronger effect on At*crk5-1* mutant hooks (Figure 6K,L) than on those of the wild type (Figure 6I,J).

The relative signal intensities are quantified in Figure 4M (PIN3-GFP), in Figure 5M (PIN7-GFP) and in Figure 6M (AUX1-YFP). Signal intensities on heat map images and relative GFP/YFP signal intensities are in good accordance.

In conclusion, the above results corroborate that the AtCRK5 protein kinase has an important role regulating PAT protein abundance and consequently auxin distribution upon hypocotyl hook formation and may directly or indirectly influence ethylene and GA3 level/sensitivity in the hook. In order to have more insights into the underlying mechanisms, gene expression and in vitro protein phosphorylation experiments were carried out.

### 2.4. Gene Expression Studies by qRT-PCR

#### 2.4.1. Expression of the Auxin Biosynthesis and Catabolism Genes without/with ACC Treatment

It is known that auxin is crucial to plant apical hook development and the PAT proteins determine the direction of the auxin flow [50]. The activity of the auxin transporter PATs is regulated at multiply levels, e.g., by transcriptionally or posttranslationally [51]. Hence, we wanted to investigate whether the less intense bending of the hypocotyl hook in At*crk5-1* mutant is the consequences of alterations in auxin biosynthesis/degradation or ethylene biosynthesis/signaling gene expressions or not. First we studied the transcript levels of a few selected genes encoding auxin biosynthesis proteins like *TRP2 (At5g54810)*, *TRP3 (At3g54640)*, *TAA1 (At1g70560)*, *AMI1, (At1g08980)*, *YUCCA3 (At1g04610)*, *NIT3 (At3g44320)*, *CYP83B (At4g31500)*, and the catabolism gene *GH3.2 (At4g27260)* pathway under control and treated (10 μM ACC) conditions. The results are presented in Figure S1. The auxin biosynthesis genes *TRP2*, *TRP3*, *TAA1*, *AMI1*, *YUCCA3*, *NIT3* and *CYP83B* showed different levels of basal expression in the two Arabidopsis backgrounds. The expression level of *TRP2*, *TRP3* and *YUCCA3* genes were slightly higher in the At*crk5-1* mutant compared to the wild type Col-0, while in the case of the other genes did not differ. Upon ACC treatment, the expression level of auxin biosynthesis genes did not alter significantly, so the exogenous ethylene did not influence the expression of the auxin biosynthesis genes. The expression of *CYP83B* gene was relatively high in both genotypes at mock and ACC treatment as well. The expression level of the *GH3.2* gene was very low and the ACC treatment did not change this. We may conclude that the expression level of the auxin metabolism genes was not affected by the absence of the AtCRK5 kinase even after ethylene application.

#### 2.4.2. Expression of the Ethylene Biosynthesis and Signaling Genes without/with ACC Treatment

Besides the auxin metabolism genes, we studied the expression level of the ethylene biosynthesis *ACS5 (At5g65800)*, *ACS7 (At4g26200)*, *ACS8 (At4g37770)* and ethylene signaling *EIN3 (At3g20770)* and *HLS1 (At4g37580)* genes in treated and control conditions (Figure S2). The ethylene biosynthesis genes displayed various but low expression levels under control conditions in both Arabidopsis backgrounds. This did not change upon ACC treatment. The expression level of the two ethylene signaling genes (*EIN3* and *HLS1*) elevated in the wild type after the ACC treatment but this increase was not significant, similarly as in the mutant background.

#### 2.4.3. Expression of the PAT Genes without/with ACC Treatment

The polar auxin transport (*AUX1*, *LAX3*, *PIN1*, *PIN3* and *PIN4*) gene expression was also analysed. The expression level of the *LAX3* and *PIN3* genes were elevated in the At*crk5-1* mutant compared to the Col-0, while the ACC treatment reduced their expression to the level of the Col-0 in control conditions (Figure S3). Interestingly, the expression level of the auxin influx transporter *AUX1* was slightly higher in the mutant, but decreased as a result of the ACC treatment. The expression level of the *PIN1* and *PIN4* remained very low upon mock and ACC treatments as well.

As a conclusion, we may say that the expression level of the auxin influx *LAX3* and auxin efflux *PIN3* genes increased and this elevation rate was down regulated by ACC treatment in the At*crk5-1* mutant to the expression level of the untreated wild type gene. We also claim that the less intense bending of the hypocotyl hook in At*crk5-1* mutant is not the consequences of the alterations in auxin biosynthesis/degradation or ethylene biosynthesis/signaling gene expressions. Probably, the altered hypocotyl hook bending phenotype of At*crk5-1* mutant is rather the consequence of posttranslational modifications (e.g., by phosphorylation/dephosphorylation events) of the auxin transporter proteins, especially of the PIN3 protein. These modifications may strongly influence the localization capability of these proteins, consequently the efficiency of the auxin flow [47,52,53,54].

### 2.5. AtCRK5 Can Phosphorylate the Auxin Efflux PIN3 Protein in vitro

**Figure 7:**
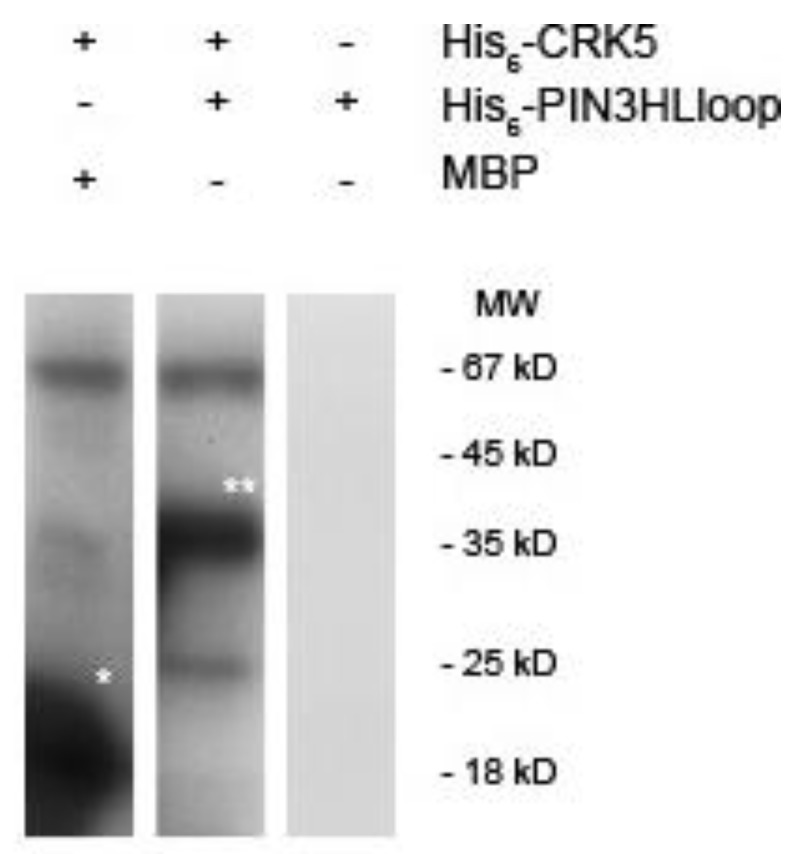
AtCRK5 phosphorylates PIN3 hydrophilic loops *in vitro.* In vitro phosphorylation assay with His6-AtCRK5 and two substrates: His6-PIN3HLloop and myelin basic protein (MBP) which was used as positive control. White asterisks indicate the phosphorylated MBP and His6-PIN3HLloop proteins, respectively.

It has already been reported that AtCRK5 phosphorylates PIN2, the main auxin efflux transporter which controls the gravitropic response of the Arabidopsis roots upon gravistimulation [42]. We, therefore, questioned whether AtCRK5 can phosphorylate other PINs. PIN3 is supposed to be the main auxin transporter during hypocotyl hook formation [10,11]. We used recombinant PIN3 fragments corresponding its full hydrophilic loop and we could demonstrate that the recombinant AtCRK5 can phosphorylate it *in vitro*. Therefore, AtCRK5, in addition to PIN2, can regulate the PIN3 auxin efflux transporter protein (Figure 7).

## 3. Discussion

### 3.1. AtCRK5 Might be a General Regulator of PIN Proteins, Auxin Distribution, and Differential Organ Growth

The sensitive shoot apical meristem of dicot plant seedlings is protected by the hypocotyl hook during their growth through the soil after germination. Shortly after the seedling erupts from the soil, the hook quickly straightens out in response to light [6,8,12]. Asymmetrical auxin accumulation at the opposite sides of the appropriate hypocotyl region is fundamental for hook formation. During the formation and maintenance phases of apical hook development, auxin distrubition was investigated by the auxin reporter DR5::GFP, which was found to be expressed at the inner side of the hook (Figure 3A,B) [6,7]. We observed that the DR5::GFP signal was less intense in the At*crk5-1* mutant than in the wild type at the inner side of the bending hypocotyl (Figure 3C-D). Accordingly, the AtCRK5 protein kinase might be required for the initial establishment of the asymmetrical auxin gradient in the hypocotyl. We have already described that the At*crk5-1* mutation retards asymmetric auxin redistribution in gravistimulated roots resulting in delayed graviresponse [42]. Therefore, AtCRK5 might be considered as a general regulator of auxin distribution during organ bending due to differential growth as demonstrated in the root [42] and the hypocotyl (this study).

The formation of the auxin gradient at the hypocotyl hook depends on the proper level, localization and cooperation of several auxin transporters. The main auxin transporters of the apical hook are the PIN (PIN-FORMED) auxin efflux carriers, namely, PIN1, PIN3, PIN4 and PIN7 [6,19], the AUX/LAX (AUXIN1/LIKE-AUX1) auxin influx carriers and the ATP-binding cassette B (ABCB) transporters [19,55]. They transport auxin from the cotyledons towards the basal side of the hypocotyl in different tissue layers during apical hook development [5,6]. PIN3 has a prominent role in hook formation and maintenance [6]. It is mainly produced at the stele and the outer side of the apical hook and transports auxin from the endodermis towards the cortex and epidermis [10,11]. The subsequent action of AUX/LAX transporters in the outer tissues results in a higher auxin drainage from the outer than the inner side of the hook establishing the auxin gradient. During the opening phase of hypocotyl emergence, this auxin asymmetry is lost due to the reduced expression of the AUX/ABCB transporters at the inner side of the hook limiting auxin accumulation in that region [11].

We found that the PIN3-GFP signal was much less intense in the At*crk5-1* mutant than in the wild type hypocotyl stele at the maintenance phase (Figure 4A–D). This indicates that the transportation of auxin by PIN3 might also be limited in the At*crk5-1* mutant at this phase of hook development. Additionally, signals of other auxin transport proteins, PIN7-GFP (auxin efflux) and AUX1::GFP (auxin influx), were also less intense in the At*crk5-1* mutant signifying overall disturbance of auxin transport in the mutant. Decreased abundance of the transporters might be ascribed either to their decreased expression and/or stability. Considering that the gene expression of neither *PIN3*, *PIN4*, nor *LAX3* was found to be significantly decreased in the mutant hypocotyl hook (Figure S3), the latter is more likely.

Having only a low level of PIN3, the formation of the asymmetric auxin gradient fails and the At*crk5-1* mutant apical hooks are not properly closed. Here we demonstrated that PIN3 can be phosphorylated by AtCRK5 in vitro (Figure 7) (see also [56]). Reduced phosphorylation of PIN3 might be responsible for its decreased stability in the mutant background. It was previously reported that AtCRK5 can also phosphorylate the hydrophilic loop of the PIN2 auxin efflux protein in vitro and the delayed gravitropic response of the At*crk5-1* mutant may reflect the defective phosphorylation of PIN2 in vivo [42]. The phosphorylation of PIN proteins is a basic requirement for their stability [52,53,54]. Subcellular PIN polarity determines directional auxin flow and therefore influences differential growth and organogenesis [17,19,20,57,58,59]. The molecular and biological function of PIN3 also requires phosphorylation [60]. The AGC kinases PID, WAG1, WAG2 and D6PK were reported to phosphorylate specific residues in the hydrophilic loops of several PINs [27,60,61]. Among them, the WAG2 protein kinase has been proposed to participate in the establishment of the asymmetrical auxin accumulation at the inner side of the hypocotyl hook where it is specifically expressed [27]. In contrast to the specific expression and localization of WAG2, AtCRK5 has been shown to be expressed in every organ of Arabidopsis [37,42]. Moreover, in the At*crk5-1* mutant, not the subcellular polarity but rather the abundance of auxin transporters, including PIN3, was affected. One may suppose that WAG2 and AtCRK5 may coordinately control the stability and subcellular localization of PIN proteins. Moreover, since we also found a delay in the gravitropic response of hypocotyls not only in the At*crk5-1* dark-grown seedlings but in the mutants of all AtCRK family members [37], we suggest that other members of this protein kinase family might also be involved in the formation of the hypocotyl hook. Presumably, the mutation of AtCRK5 has only a limited effect on PIN abundance and hypocotyl bending due to the presence of the other PIN-phosphorylating kinases having somewhat overlapping functions with AtCRK5.

### 3.2. The AtCRK5 Kinase Influences the Hormonal Crosstalk Regulating Hypocotyl Hook Development

The plant hormone ethylene is known to control apical hook development by upregulating the expression of the auxin biosynthesis gene *TAR2* and fine tuning asymmetric auxin distribution among others via the HOOKLESS1 (HLS1) N-acetyltransferase [2,5,10,11,13]. Exaggerated hook bending is observed upon exogenous ethylene application [22,23], while deficiencies in ethylene signaling prevent hook formation [5,6]. We found that upon treatment by the ethylene precursor ACC, the transition between the formation and maintenance phases was delayed (Figure 1B). This resulted in exaggerated hook bending with up to 240° hook angle for wild type and up to 200° for the mutant (Figure 1A). Both wild type and mutant hooks treated with the ethylene precursor ACC had stronger DR5::GFP signals at the inner region of the hooks unlike the untreated samples indicating the positive effect of ethylene on the auxin gradient in both cases (Figure 3E-H). Similarity of the ethylene response of the *Atcrk5-1* mutant to the wild type was supported by similar ethylene-responsive expression of the auxin-related *TAA1* (close homologue of *TAR2*) and *HLS1* genes in both genotypes (Figure S1 and Figure 2). Our investigation of the PIN3 and AUX1 polar auxin transporter distributions at the hypocotyl hooks revealed that the PIN3, PIN7 and AUX1 levels - based on GFP signal intensity - were elevated upon exogenous ethylene treatment (Figure 4E–H; Figure 5E–H; Figure 6E-H). This is in good accordance with the results of [13], namely that exogenous ethylene is able to broaden the auxin gradient at the hypocotyl hook. Our data also indicate that both the wild type and At*crk5-1* mutant seedlings responded to exogenous ethylene by increased auxin transporter protein stability resulting in enhanced auxin accumulation at the inner side of the hook. Evidently, exogenous ethylene compensated for the restricted hook formation of the At*crk5-1* mutant (Figure 1A), however there was no difference between the mutant and the wild type considering the expression of ethylene synthesis/signaling genes at the maintenance phase suggesting that the ethylene synthesis rate is also similar in the two genotypes at this stage (Figure S2).

We could determine however, a significantly lower level of gibberellin in the hypocotyl of the At*crk5-1* mutant at the time of hook maintenance (Figure 2B). Although it indicates that the AtCRK5 kinase may directly regulate gibberellin synthesis, we suppose that the reduced gibberellin level is an indirect effect of the diminished auxin accumulation in the hypocotyl. Auxin was shown to form a positive feed-back loop with gibberellin during hypocotyl development enhancing the expression of gibberellin biosynthesis genes [30,31].

Gibberellins are fundamental plant hormones for many developmental processes and can modulate both auxin and ethylene concentrations during apical hook development [10,11,21,62,63]. GAs – together with ethylene - are essential to maintain the apical hook in the closed form during seedling emergence from the soil [10,11]. Seedlings treated by GA biosynthesis inhibitors have decreased expression of DR5::GFP at the inner side of the apical hook [7], similarly to the DR5::GFP pattern found in the apical hook of At*crk5-1* during skotomorphogenesis (Figure 3I-J). Additionally, active GAs is necessary to maintain the expression of PIN3 and PIN7 in the apical hook and the *pin3pin7* knockout mutant is resistant to exogenous GA treatment [7]. The Arabidopsis gibberellin biosynthesis mutant *ga1* which is impaired in an early step of GA biosynthesis is also impaired in auxin transport [64,65]. The diminished auxin transport did correlate with the reduction of the abundance of PIN auxin efflux transporters in *ga1* but exogenous GA treatment could restore the PIN protein levels to those of the wild type [66]. Exogenous GA_3_ increased and broadened the expressions of the main auxin efflux and influx transporters PIN3, PIN7 and AUX1 in the apical hooks of the wild type as well as the At*crk5-1* mutant seedlings (Figure 4I–L; Figure 5I–L; Figure 6I–L) and in this latter genotype the hook angle was restored to the normal level during the maintenance phase (Figure 2A). Interestingly, we found increased *PIN3* and *AUX1/LAX3* expression in the At*crk5-1* hypocotyls in comparison to the wild type (Figure S3). Since At*crk5-1* hypocotyls have lower level of GAs (Figure 2B), this observation seems to disagree with the requirement of gibberellin for *PIN3/PIN7* expression. We presume that the absence of AtCRK5 protein kinase leads to the destabilization of auxin transporters leading to their degradation in the At*crk5-1* mutant. The increased transcription of *PIN3* and *LAX3* might represent a kind of compensation response, but the still limited auxin transport is insufficient to establish the proper auxin gradient at the hypocotyl hook. This results in the restricted bending (closure) of the hypocotyl tip. However, exogenous GA_3_ can ameliorate the auxin gradient in the mutant either via augmenting the expression of the auxin transporters or increasing their stability. This view is supported by the increased accumulation (fluorescence) of PIN3-GFP, PIN7-GFP and AUX1::GFP upon GA_3_ treatment of the hypocotyls (Figure 4C–D,K–L; Figure 5C–D,K–L; Figure 6C–D,K–L).

**Figure 8:**
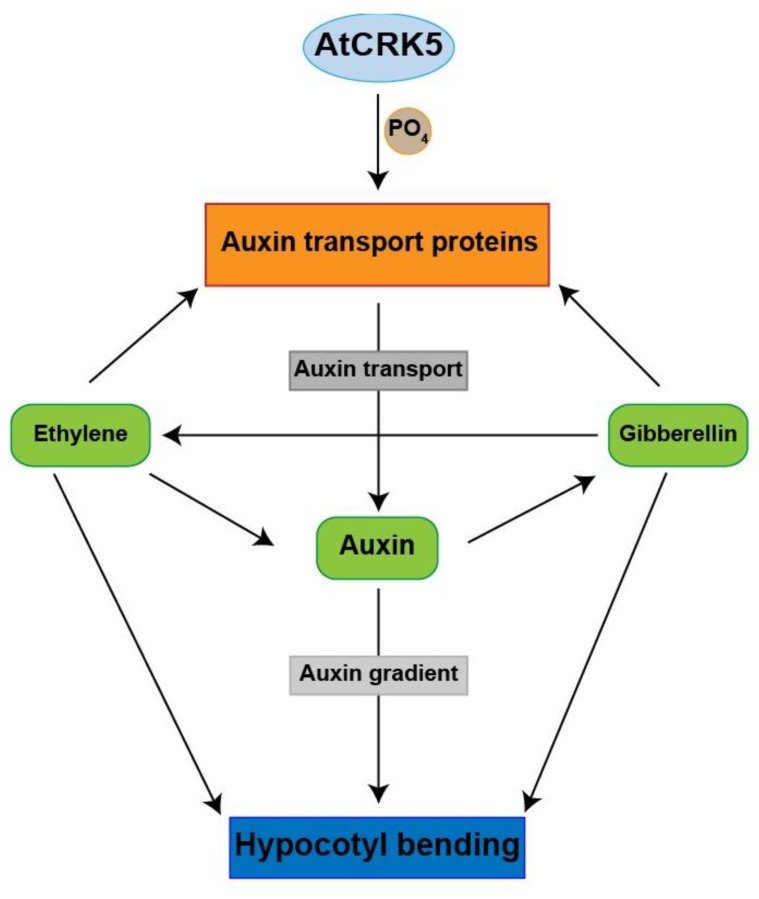
Hypothetical role of the AtCRK5 protein kinase in the regulation of hypocotyl bending during skotomorphogenesis in *Arabidopsis thaliana*. AtCRK5 is proposed to affect the auxin homeostasis increasing the stability of auxin transporter proteins and thus contributing to auxin transport efficiency and gradient establishment. The increased auxin level at the inner side of the hook is in a feedback loop with ethylene and gibberellin synthesis/signaling and their coordinated and balanced function is required for hypocotyl bending. Absence of the kinase hinders the establishment of the proper auxin gradient due to lowered auxin transporter stability. The lower level of auxin and/or its altered distribution feeds back to ethylene and GA signaling/synthesis and a new hormonal balance is established at a shallow auxin gradient resulting in limited hypocotyl closure but proper timing of its development.

Here we claim that we extended the known functions of the AtCRK5 protein kinase, because it regulates the formation of the asymmetrical auxin gradient in the hypocotyl hook during skotomorphogenesis in *Arabidopsis thaliana*. Therefore, AtCRK5 can be considered as a regulator of apical hook establishment. Our hypothetical model for the regulatory role of the AtCRK5 protein kinase during hypocotyl hook development in relation to auxin-ethylene-GA crosstalk is presented in Figure 8. According to this model, the AtCRK5 kinase enforces the stabilization of auxin transporters at the time of hypocotyl hook establishment. This is required for the formation of a steep auxin gradient and a complete hypocotyl hook closure. Absence of the kinase results in lowered auxin transporter stability, limited establishment of the auxin gradient, lower level of auxin accumulation at the inner side of the hook, and limited closure. Due to the feedback regulation between auxin and gibberellins, the gibberellin level is also lowered in the hook that further contributes to the limited synthesis of auxin transporters. Nevertheless, due to the regulatory circuit including auxin, ethylene, and GAs, the timing of the hook developmental phases is maintained in the mutant but at lower levels of endogenous plant hormones still functioning in a coordinated way.

## 4. Materials and Methods

### 4.1. Plant Material and Growth Conditions

All plants used in this study were in *Arabidopsis thaliana* (L.) Columbia-0 ecotype (Col-0) background. The following lines were used in this study: the At*crk5-1* mutant has been described previously in [37,42], the auxin inducible DR5::GFP [46], the PIN3:PIN3-GFP [6], PIN7:PIN7-GFP [48] and AUX1::YFP [49] constructs were also described in [42]. All the PAT proteins were driven by their own genomic promoters. We introduced the DR5::GFP and PIN7:PIN7-GFP into wild type (Col-0) and mutant (At*crk5-1)* backgrounds via deep floral transformation [42,67]. PIN3:PIN3-GFP and AUX1::YFP were introduced into wild type and mutant backgrounds by sexual crossings [42]. For seed germination and hook kinetic measurements, wild type and mutant seeds were sterilized and kept at 4 °C for two days as indicated in [42]. After it, imbibed seeds were transferred onto plates containing ½ strength Murashige and Skoog medium (MS) with 0.5% sugar, 0.8% agar, pH: 5.7 (Duchefa Biochemie, Haarlem, The Netherlands). After seed transfer (AST), the plates were kept in white light for 5 h to stimulate and synchronize seed germination. Then plates were kept vertically in dark for the process of germination for 4-7 days at 22 °C. The basic media was supplemented with 10 μM 1-aminocyclopropane-1-carboxylic acid (ACC, Sigma-Aldrich, Merck KGaA, Darmstadt, Germany) or 1 μM gibberellic acid (GA_3,_ Sigma Chemical Co., St. Louis, MO, USA) where it is indicated. The ACC and GA_3_ were dissolved in DMSO:methanol solution (1:1) and stock solutions were prepared at 10 mM (ACC) and 1 mM (GA_3_) concentrations for further use.

### 4.2. Time Lapse Assays

Rates of hypocotyl hook formation were scored after radicle appearance for 4–7 days during formation (0–54 h after seed transfer [AST]), maintenance (54–94 h AST) and opening (94–170 h AST) phases of germination. Developing seedlings were photographed in every 6 h using a camera (Canon PC1438, Canon Inc., Japan) under green safety light (520 nm led light, home prepared lamp, Freiburg, Germany) to avoid triggering photomorphogenesis. Hook angle between the hypocotyl axis and cotyledons (hook curvatures) were measured on the photographs by the ImageJ software (NIH, Bethesda, MD, USA). The angles were defined according to the inset in Figure 1. At least 100 wild-type and mutant seedlings were finally monitored in three biological repeats. Student’s t-test was used for statistical analysis for all quantitative measurements.

### 4.3. Total GA Measurement by Competitive GAs Elisa Assay

For ELISA measurement, we followed the protocol instructions of the kit (ELISA Kit for Gibberellic Acid (GA), CEA759Ge, Cloud Clone Corp. Wuhan, China). We measured the total GA content in dried seeds and seedlings. From dried seeds, 100 mg was used from the wild type (Col-0) and the mutant (At*crk5-1*). The GA content was directly measured in these seeds as the zero-time point. In addition, wild-type and mutant seeds were germinated on ½ MS media using nylon mesh in the dark after five-hour light induction. Seedlings germinated in complete dark were collected at 48 h and 60 h after seed transfer under safety green light condition into Eppendorf tubes and the samples were stored in liquid nitrogen. The seedlings from these experiments were grounded under liquid nitrogen using a mortar and pestle with small amount of quartz sand plus 10-10 mL of 100% of methanol. In the case of the dry seeds, we added 1ml of water and 10mL of methanol for the grinded dry seeds samples. Extracts were put into 15 mL centrifuge tubes and then incubated o/n at 4 °C on shaker. Cellular debris was removed by centrifugation (17000× *g*, 10 min). The supernatants were divided equally into new tubes and lyophilized o/n to complete dryness. 200 μL PBS was added to each sample and resuspended carefully, and then we followed the kit instruction. Two biological repeats were performed for total GA content evaluation.

### 4.4. RNA Isolation and Real Time Quantitative PCR (qRT PCR) for Hypocotyl Gene Expression

Isolation of RNA was performed from 100 mg material collected from 3-days-old wild-type (Col-0) and mutant (At*crk5-1*) Arabidopsis seedlings without and with 10 μM ACC treatment. The isolation was performed by TRI reagent (Sigma-Aldrich) [68]. Total RNA was DNase-treated with TURBO DNA-free™ Kit (Invitrogen by Thermo Fisher Scientific, Vilinus, Lithuania) and cDNA synthesis of 1 μg of total RNA was carried out in a 20 μL reaction volume using RevertAid M-MuLV Reverse Transcriptase according to the supplier’s recommendation (Applied Biosystems, Thermo Fischer Scientific, Vilinus, Lithuania) using random hexamers. Quantative Real-time PCR (qRT-PCR) was carried out using the SYBR Green master mix (Applied Biosystems, Thermo Fischer Scientific) by ABI 7900 Fast Real Time System (Applied Biosystems) using the following protocol: 45 cycles at 95 °C for 15 s, followed by 60 °C for 1 min. The normalized relative transcript levels were obtained by the 2^−ΔΔCt^ method [69]. Reactions were made in triplicates and minimum two independent biological repetitions were performed. *GAPDH2 (AT1G13440)* was used as an endogenous control. All set of qRT PCR primers used in this study are listed in Table S1.

### 4.5. PINs-GFP Protein Abundance Monitoring in Hypocotyl Hooks by LSM Microscopy

Hypocotyl hooks of the 3 days old dark grown wild type Col-0 and At*crk5-1* seedlings expressing the DR5::GFP, PIN3:PIN3-GFP, PIN7:PIN7-GFP and AUX1::GFP constructs were imaged under green safety light using Olympus FV1000 confocal laser scanning microscope (Tokyo, Japan) as described in [42]. We used always the same microscopic parameters within the corresponding experiments. Expression pattern in minimum 10 seedlings from each independent GFP/YFP tagged line was monitored in two different experiments. Generally, the whole seedlings and/or hypocotyl hooks were optically sliced (10 slices per seedlings) with LSM microscope using 10x magnification. Finally, Z-stack images were presented in the Figures. Color-coded heat maps were created to visualize the fluorescence intensity differences in hypocotyl hooks [42]. These images were prepared using the Adobe Illustrator software.

To quantify the fluorescence signal intensities, Z-stack images were used from each construct. For this quantitative analysis an equal squared area (regions of interest) were designed on the images. Fluorescence intensity was measured using Olympus FV1000 software (Tokyo, Japan). The DR5::GFP signal intensities were measured at the concave side of the hooks of wild-type (Col-0) and mutant (At*crk5-1*) treated with or without chemicals ACC and GA_3,_ respectively. The PIN3:PIN3-GFP and AUX1::YFP signal abundance was quantified between the convex and concave sides of the wild type and mutant hooks. The relative intensities of PIN7:PIN7-GFP signals between the wild type and mutant seedlings were measured along the hypocotyls. In all experiments, at least 10 seedlings from wild type and mutant categories were investigated. All quantitative data was statistically analyzed using the Student’s *t*-test. Experimental data were obtained from minimum two independent biological repeats.

### 4.6. In vitro Kinase Assay

#### 4.6.1. PIN hydrophilic Loop Region Cloning

For cloning of the hydrophilic (HL) loop of the PIN3 auxin efflux protein into a bacterial protein expression vector, we amplified the corresponding cDNA sequence from *Arabidopsis thaliana* cDNA using the high fidelity Phusion polymerase (Thermo Fischer Scientific, Lithuania) following the manufacturer’s instructions. After amplification, the BamHI-EcoRI enzyme-digested cDNA fragment was ligated into the pBluescript II SK plasmid. Sequencing verified that the cloned fragment is error free. The PIN3HL loop fragment was moved into the pET28A (Novagen part of Merck KGaA, Darmstadt, Germany) protein expression vector, to get in frame fusion with the N-terminal 6XHis tag.

#### 4.6.2. Purification of 6XHis Tagged Protein

For protein expression studies, we transformed the pET28c-CRK5 [42] and the pET28a-PIN3HL constructs into BL21DE3Rosetta competent cells, then streaked them onto LB media supplemented with 25 mg/l kanamycin, 34 mg/l chloramphenicol and 1% of glucose, and incubated the plates o/n at 37°C. For protein expression, purification, and dialysis we follow the procedure as in [42]. Briefly: inoculation was performed from the plate into 25 mL of LB liquid media supplemented with antibiotics as before plus 1% glucose. Cultures were diluted with fresh LB media at 1:10 ratio, and then they were incubated on a shaker at 37°C for 2 h. When the OD600 reached 0.6-0.8, IPTG was added at final concentration of 1mM. The growth was continued for 4–5 h at room temperature to complete the protein induction. The induced bacteria were collected by centrifugation and kept at -20 °C until use. His6-CRK5 and His6-PIN3HL proteins were purified by Ni-NTA agarose affinity chromatography following the manufacturer’s instructions (Novagen part of Merck KGaA, Darmstadt, Germany). After elution with 100-200 mM imidazole, we checked the fractions by 10% SDS-polyacrylamide gel electrophoresis (SDS-PAGE). The fractions containing most of the His6-CRK5 or His6-PIN3HL proteins were identified, pooled, and dialyzed (10 mM Tris-HCl (pH 7.5), 50 mM NaCl, 10% glycerin and 5 mM 2-mercaptoethanol) at 4 °C and stored at −80 °C for later use.

#### 4.6.3. In vitro Kinase Assays

The in vitro kinase assays were carried out with 1 μg His6-CRK5 kinase in 20 μL kinase buffer (20 mM Tris-HCl [pH 8.0], 5mM MgCl2, 1mM DTT and 5µCi [γ-32P]ATP) containing 5μg Myelin Basic Protein (MPB) (Sigma, Germany) as a control kinase substrate, or His6-PIN3HL as substrate room temperature for 30–45 min. The reaction stopped by adding 1× Laemmli SDS sample buffer, boiled and then size separated by 10% SDS-PAGE. After staining with Coomassie dye, the gel was subjected to autoradiography using X-ray film.

### 4.7. Bioinformatics Analysis

Primer preparation for genes investigated in this study were constructed using Primer3Plus software (http://www.bioinformatics.nl/cgi-bin/primer3plus/primer3plus.cgi. ABI SDS software (Applied Biosystems, Foster City, CA, USA) was used to analyze the specificity of the amplifications of the genes for expression by qRT PCR. Hypocotyl hook bending was measured by ImageJ software (https://imagej.net/Fiji/Downloads). Average fluorescence intensity was measured using Olympus FV1000 software (Tokyo, Japan).

### 4.8. Accession Numbers

Sequence data used in this study can be found in the Arabidopsis Information Resource (TAIR) and GenBank (NCBI) databases under the following accession numbers: *CRK5 (At3g50530), PIN1 (At1g73590)*, *PIN3 (At3g70940)*, *PIN4 (At2g01420)*, *PIN7 (At1g23080)*, *AUX1 (At2g38120), LAX3 (At1g77690)*, *TRP2 (At5g54810)*,*TRP3 (At3g54640)*, *YUCCA3 (At1g04610)*, *AMI (At1g08980)*, *TAA1 (At1g70560)*, *CYP83B1 (At4g31500)*, *NIT3 (At3g44320)*, *GAPDH2 (At1g13440)*, *GH3.2(At4g27260)*, *EIN3 (At3g20770)*, *HLS1 (At4g37580)*, *ACCSynthase5 (At5g65800)*, *ACCSynthase7 (At4g26200)*, *ACCSynthase8 (At4g37770)*.

## Supplementary Materials

Supplementary materials can be found at https://www.mdpi.com/1422-0067/20/14/3432/s1.
