# Supplementary material for: AtCRK5 Protein Kinase Exhibits a Regulatory Role in Hypocotyl Hook Development during Skotomorphogenesis
Source: Int J Mol Sci. 2019 Jul 12;20(14):3432. doi: 10.3390/ijms20143432 (PMC6678082; doi:10.3390/ijms20143432)
Supplement: Supplementary file 1 [file ijms-20-03432-s001.zip › supplementary figures.docx]

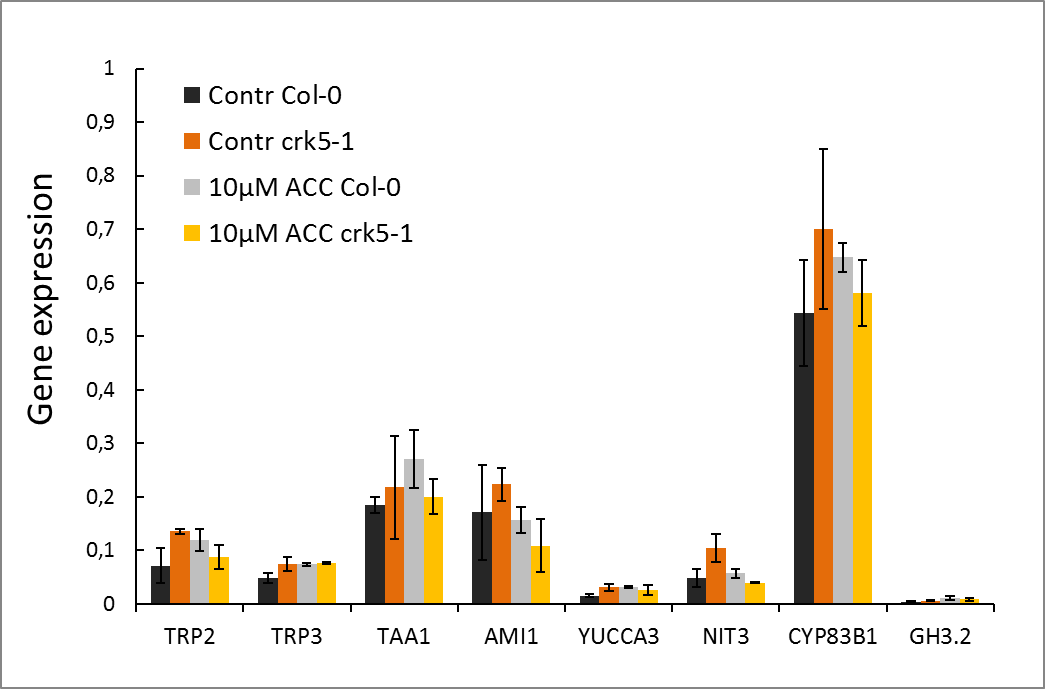


**Figure S1. Influence of the ACC treatment on gene expression.** Expression of the auxin biosynthesis (*TRP2*, *TRP3*, *TAA1*, *AMI1*, *YUCCA3*, *NIT3*, *CYP83B*) and catabolism (*GH3.2*) genes, without and with 10 μM ACC treatments from 3 days old wild type (Col-0) and mutant (At*crk5-1*) *Arabidopsis* seedlings. As an endogenous control *GADPH2* was used. SE values arised from two independent experiments.


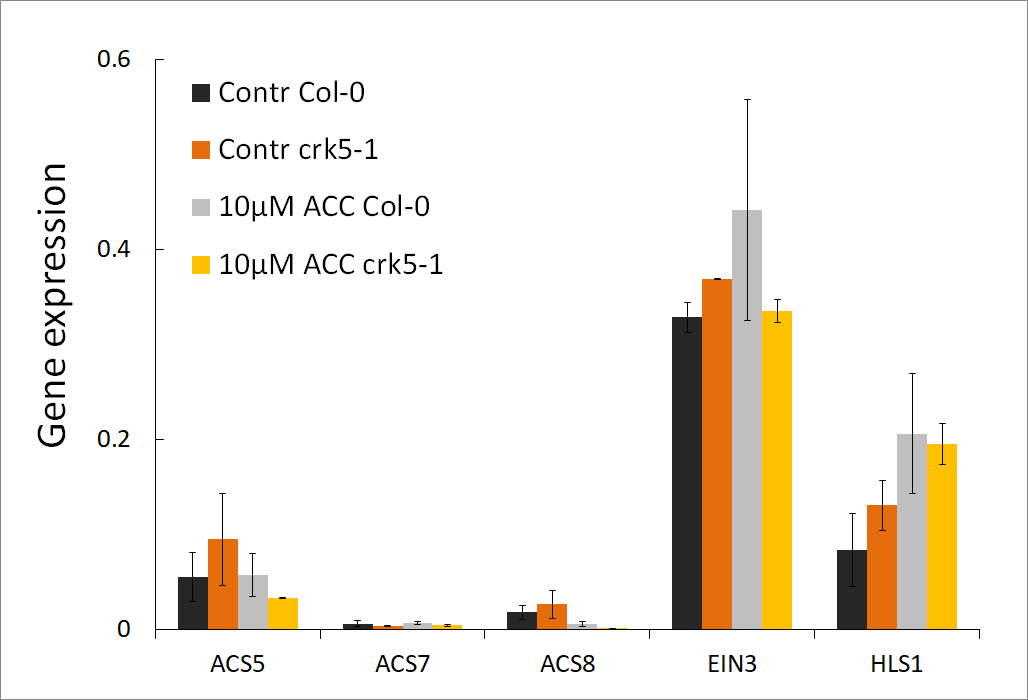


**Figure S2. Gene expression upon ACC treatment.** Expression of the ethylene biosynthesis (*ACS5*, *ACS7* and *ACS8*) and ethylene signaling (*EIN3*, *HLS1*) genes were measured by qRT-PCR without and with 10 μM ACC treatments from 3 days old wild type (Col-0) and mutant (At*crk5-1*) *Arabidopsis* seedlings. As an endogenous control *GADPH2* was used. SE values arised from two independent experiments.


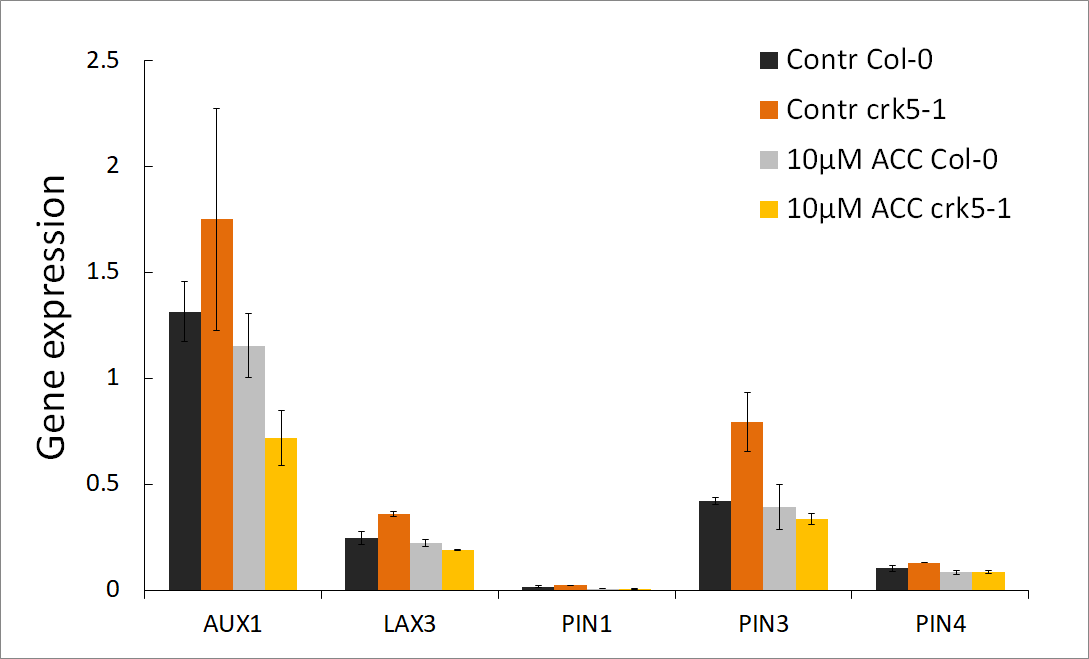


**Figure S3.** Gene expression upon ACC treatment. Expression of the polar auxin transport (*AUX1*, *LAX3*, *PIN1*, *PIN3* and *PIN4*) genes were measured by qRT-PCR without and with 10 μM ACC treatments from 3 days old wild type (Col-0) and mutant (At*crk5-1*) *Arabidopsis* seedlings. As an endogenous control *GADPH2* was used. SE values arised from two independent experiments.

**Supplemental Table 1.** List of the primers used in this study.
